# Diffuse ileal ganglioneuromatosis mimicking a gastrointestinal stromal tumor: A case report

**DOI:** 10.1097/MD.0000000000016305

**Published:** 2019-07-05

**Authors:** Xiaonan Yin, Xin Chen, Renjia Shu, Chaoyong Shen, Yuan Yin, Zhaolun Cai, Jian Wang, Zhou Zhao, Huijiao Chen, Bo Zhang

**Affiliations:** a Department of Gastrointestinal Surgery, West China Hospital, Sichuan University, Chengdu, Sichuan, China; b West china school of public health, Sichuan University, Chengdu, Sichuan, China; c Department of Pathology, West China Hospital, Sichuan University, Chengdu, Sichuan, China.

**Keywords:** differential diagnosis, diffuse intestinal ganglioneuromatosis, gastrointestinal stromal tumor

## Abstract

**Rationale:**

Intestinal ganglioneuromatosis (IGNM) is a rare disease, defined by an abnormal proliferation of ganglion cells, nerve fibers and Schwann cells in the enteric nerve system.

**Patient concerns:**

A 54-year-old woman presented with a one-year history of recurrent episodes of hypogastric pain, with vomiting, nausea, melena, and weight loss of 10 kg in recent 5 months.

**Diagnoses:**

The patient was diagnosed as a diffuse IGNM by pathological examination.

**Interventions:**

A complete excision of the tumor was performed.

**Outcomes:**

On follow-up after 26 months, the patient was asymptomatic without complications.

**Lessons:**

This report showed a rare case of diffuse IGNM not associated with NF1 or MEN2b. Preoperative radiological examination suggested an intestinal GIST, yet the final diagnosis of diffuse IGNM was made according to the pathological examination of the resected specimen. Although the prevalence of ganglioneuromatosis is low, this condition should be considered in the differential diagnosis of intestinal mass in adults.

## 1 Introduction

Intestinal ganglioneuromatosis (IGNM) is a rare benign tumor characterized by proliferation of ganglion cells, nerve fibers and supporting cells of enteric nervous system.^[1]^ They may be discovered in any age and develop sporadically or in association with syndromes such as multiple endocrine neoplasia (MEN) type 2b and neurofibromatosis type 1 (NF1).^[2,3]^ IGNM may occur in 3 forms: as a single polyp, as multiple polyps (ganglioneuromatous polyposis) and as diffuse involvement of the intestinal wall (diffuse ganglioneuromatosis).^[4]^ Single polypoid ganglioneuromatosis are not typically associated with NF1 or MEN2b. However, ganglioneuromatous polyposis and diffuse ganglioneuromatosis often occur in association with NF1, MEN2b, juvenile polyposis, Cowden disease, and non-familial adenomatous polyposis. Its occurrence in the absence of the above-mentioned syndromes is extremely rare.

Diffuse ganglioneuromatosis has 2 variants: mucosal or transmural. In children, both mucosal and transmural variants can be seen, but in adults the most frequently variant is the mucosa.^[5]^ This paper reports a transmural diffuse ileal ganglioneuromatosis in an adult, unassociated with any manifestation of MEN2b, NF1, etc. Preoperative diagnosis suggested a gastrointestinal stromal tumor, yet the final diagnosis defined a diffuse ganglioneuromatosis depended on the pathological examination of the resected specimen.

## 2 Case presentation

**Figure 1:**
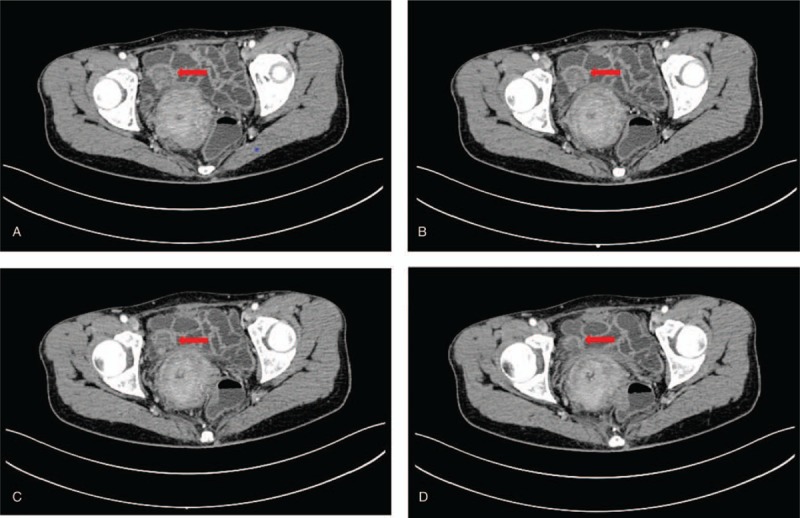
Abdominal enhanced computed tomography showed a soft tissue mass shadow with mild delayed enhancement extended into the distal ileal lumen (A, B, C, D). The narrow indicates the tumor.

A 54-year-old female presented in our department in July 2016 with a one-year history of recurrent episodes of hypogastric pain. She stated that the worsening abdominal pain associated with vomiting, nausea, melena, and weight loss of 10 kg in recent 5 months. She was previously healthy and had no special medical history. Regarding her family history, there were no other cases of gastrointestinal tumors, inflammatory bowel diseases. No physical and laboratory abnormalities were found. Upper gastrointestinal endoscopy and colonoscopy were all normal. An abdominal enhanced computed tomography (CT) showed a 2.4 × 2.3 cm soft tissue mass shadow with mild delayed enhancement extended into the distal ileal lumen of the right pelvic (Fig. 1); an intestinal gastrointestinal stromal tumor (GIST) was suspected.

**Figure 2:**
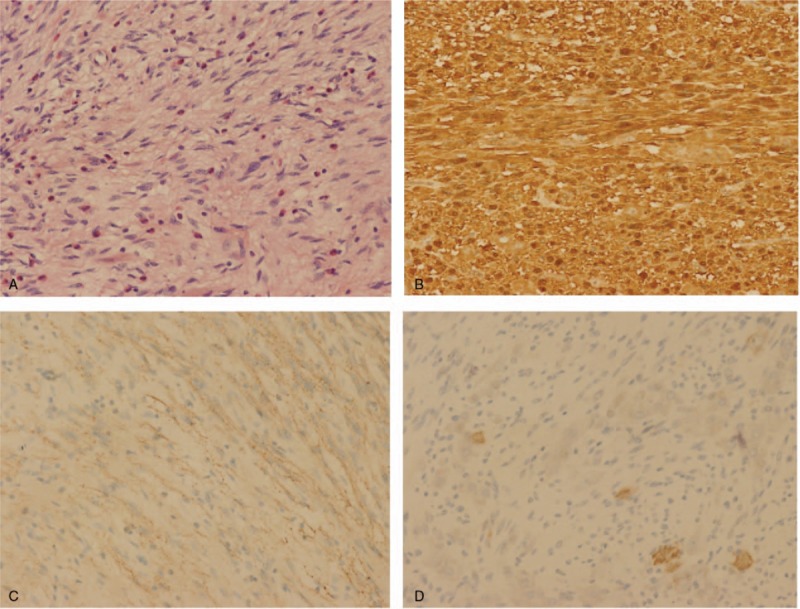
A. Proliferation of spindle cells mixed with mature ganglion cells (HE, ×200); B. Intense and diffuse expression with immunohistochemical stain S-100 protein (×200); C. Strong immunoreactivity for synaptophysin (×200); D. Strong positivity with immunohistochemical stain NeuN protein (×200).

**Figure 3:**
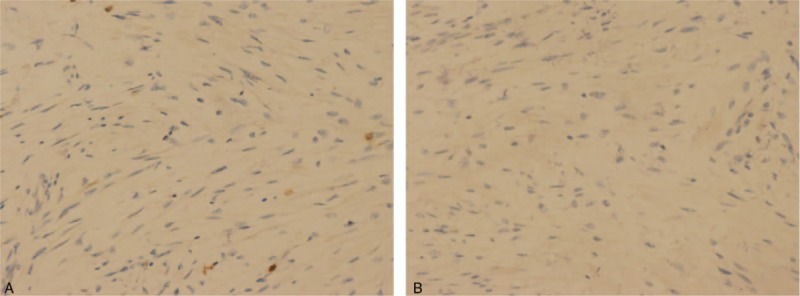
Immunohistochemical analysis demonstrated that the tumor cells were negative for CD117 (×200), and DOG-1 (×200).

Open surgery disclosed that a palpable mass, about 2 cm in diameter, located in the ileum 320 cm from the ieocaecal valve. The tumor manifested as a transmural mass extending to the ileal lumen which was adjacent to the right pelvic side wall, resulting in the narrow of lumen and the dilatation of the ileum proximal to the stenosis. These findings were consensus with the CT finding. A complete excision of the tumor was performed. Histologic examination revealed a diffuse infiltration of the muscularis propria by a proliferation of spindle cells and mature ganglion cells. Immunohistochemical staining showed that strong immunoreactivity for S-100 protein, synaptophysin, and NeuN (Fig. 2); Immunostaining for anti-CD117 and DOG-1 for interstitial cells of Cajal was negative, and positive Ki67 <1% (Fig. 3). Thus, a final pathologic diagnosis of diffuse intestinal ganglioneuromatosis (IGNM) was made.

A thorough physical examination excluded the association of IGNM with systemic syndromes, namely NF-1and MEN2b. Following her diagnosis of sporadic IGNM, the patient was asymptomatic without complications after 26 months of follow-up.

## 3 Discussion

Intestinal ganglioneuromatosis (IGNM) is a rare disease, defined by an abnormal proliferation of ganglion cells, nerve fibers, and Schwann cells in the enteric nerve system. This disease can be found in any part of the gastrointestinal tract. IGNM is equally distributed between males and females and is more frequently seen in children.^[6]^ The clinical presentation of IGNM is variable and depends on the location and extent of the lesions, and their effect on gastrointestinal motility. The prevalent symptoms in IGNM are abdominal pain, constipation or diarrhea, bleeding, changes in bowel habits, and intestinal obstruction due to stricture formation.^[7,8]^

In 1994, Shekita and Sobin^[9]^ classified IGNM into 3 types: polypoid ganglioneuroma, ganglioneuromatous polyposis, and diffuse ganglioneuromatosis. The first type, polypoid ganglioneuroma occurs as solitary or a few small polyps presenting with sessile or pedunculated morphology. The lesions are mucosal or submucosal and are mostly seen in the large intestine. Patients with polypoid ganglioneuroma are not associated with NF1, MEN2b or other genetic syndromes.^[10]^ The second type, ganglioneuromatous polyposis is characterized by numerous (more than 20) mucosal and/or submucosal polyps with a sessile or pedunculated form, and is predominately located in the colon and terminal ileum.^[11]^ Ganglioneuromatous polyposis can be isolated or associated with one of the following syndromes: MEN2b, NF1, Cowden disease, non-familial colic polyposis or juvenile polyposis. Gastrointestinal ganglioneuromatosis can be the first presentation of these syndromes.^[12]^ The third type, diffuse ganglioneuromatosis is disseminated intramural or transmural lesions characterized by hyperplasia of the myenteric plexus. The most frequently involved location is the terminal ileum, colon and appendix. It can be solitary or associated with MEN2b, NF1, juvenile polyposis, tuberous sclerosis, Cowden disease, etc.^[13]^ However, the patient in our case had an isolated IGNM without any systemic syndromes or familial diseases. Diffuse ganglioneuromatosis has 2 froms, mucosal or transmural. In children, both variants can be found and are highly associated with RET-proto-oncogene abnormalities, while in adults the disease mostly involves the mucosa, and genetic abnormalities is not mandatory. The case reported in this paper is transmural.

Due to the unspecific clinical presentations and imaging manifestations, the defined diagnosis of IGNM is very difficult. Some differential diagnoses that can involve the gastrointestinal tract were proposed, including intestinal tuberculosis, Crohn disease, intestinal tumors (adenocarcinoma, GIST, leiomyoma, lymphoma, neurofibroma, schwannoma), cytomegaly virus infection, NSAIDs enteropathy and amyloidosis.^[13]^ In our case, abdominal enhanced CT revealed a soft tissue mass in the enteric cavity, leading to luminal narrowing. An intestinal stromal tumor was suspected based on CT image and clinical manifestation. However, pathological examination defined a diffuse IGNM. Overall, it is difficult to differentiate GIST and IGNM before operation. First, Diffuse ileal ganglioneuromatosis (IGNM) and gastrointestinal stromal tumor (GIST) shared similar clinical presentations, such as abdominal pain, bleeding, constipation or diarrhea, intestinal obstruction, and changes in bowel habits. Second, the imaging features in IGNM and GIST are similar, which manifested as submucosal soft tissue masses in CT image. Hence, the final diagnosis depended only on the pathological examination. Generally, conservative treatment is invalid for diffused IGNM, and complete resection is needed in symptomatic cases. Because this disease is highly associated with NF1 or MEN2b, screening for associated clinical features such as external stigmata, skin nodule, multiple café-au-lait spots, and tumors at other sites such as breast, uterus, thyroid and colon, are recommended.

## 4 Conclusion

This report showed a rare case of diffuse IGNM not associated with NF1 or MEN2b. Preoperative radiological examination suggested an intestinal GIST, yet the final diagnosis of diffuse IGNM was made according to the pathological examination of the resected specimen. Although the prevalence of ganglioneuromatosis is low, this condition should be considered in the differential diagnosis of intestinal mass in adults.

## Acknowledgments

The authors thank the patient who kindly agreed to provide her with the data we used in this case. The authors gratefully acknowledge the doctors working together for their support.

## Author contributions

**Conceptualization:** Zhou Zhao, Huijiao Chen, Bo Zhang.

**Data curation:** Chaoyong Shen, Yuan Yin.

**Formal analysis:** Xiaonan Yin, Xin Chen.

**Funding acquisition:** Bo Zhang.

**Investigation:** Renjia Shu, Jian Wang.

**Methodology:** Chaoyong Shen, Yuan Yin.

**Resources:** Zhaolun Cai.

**Software:** Zhaolun Cai.

**Supervision:** Renjia Shu.

**Visualization:** Jian Wang, Zhou Zhao.

**Writing – original draft:** Xiaonan Yin, Xin Chen.

**Writing – review & editing:** Huijiao Chen, Bo Zhang.

Bo Zhang orcid: 0000-0002-0254-5843.

## References

[1] Haggitt RC Reid BJ Hereditary gastrointestinal polyposis syndromes. Am J Gastroenterol 1988;83:183–6. 3024515 10.1097/00000478-198612000-00006

[2] Barwick KW Gastrointestinal manifestations of multiple endocrine neoplasia, type IIB. N Engl J Med 1983;308:635–9. doi: 10.1056/NEJM198303173081106. 6132946 10.1097/00004836-198302000-00018

[3] Fuller CE Williams GT Gastrointestinal manifestations of type 1 neurofibromatosis (von Recklinghausen's disease). Cornea 1991;10:454–9. 1916682 10.1111/j.1365-2559.1991.tb00888.x

[4] Shekitka KM Sobin LH Ganglioneuromas of the gastrointestinal tract. Relation to Von Recklinghausen disease and other multiple tumor syndromes. Dermatol Clin 1995;13:99–103. 7906923

[5] Chambonniere ML Porcheron J Scoazec JY Intestinal ganglioneuromatosis diagnosed in adult patients. Gastroenterol Clin Biol 2003;27:219–24. Epub 2003/03/27. PubMed PMID: 12658132. 12658132

[6] Fortea-Sanchis C Gomez-Quiles L Diffuse intestinal ganglioneuromatosis in the adult. Cir Esp 2015;93:665 doi: 10.1016/j.ciresp.2015.06.010. Epub Oct 1. 26427303 10.1016/j.ciresp.2015.06.010

[7] Charagundla SR Levine MS Torigian DA Diffuse intestinal ganglioneuromatosis mimicking Crohn's disease. Eur J Pediatr Surg 2004;14:384–91. doi: 10.1055/s-2004-821120. 15100112 10.2214/ajr.182.5.1821166

[8] Rosenfeld EH Chumpitazi BP Castro E Diffuse intestinal ganglioneuromatosis causing severe intestinal dysmotility in a child with a PTEN mutation. J Pediatr Gastroenterol Nutr 2019;68:e35–7. 29927861 10.1097/MPG.0000000000002072

[9] Shekitka KM Sobin LH Ganglioneuromas of the gastrointestinal tract. Relation to Von Recklinghausen disease and other multiple tumor syndromes. Am J Surg Pathol 1994;18:250–7. Epub 1994/03/01. PubMed PMID: 7906923. 7906923

[10] Chan OT Haghighi P Hamartomatous polyps of the colon: ganglioneuromatous, stromal, and lipomatous. World J Gastroenterol 2006;12:7874–7. 17090203 10.5858/2006-130-1561-HPOTCG

[11] Fiori E Pozzessere C Lamazza A Endoscopic treatment of ganglioneuroma of the colon associated with a lipoma: a case report. J Med Case Rep 2012;6:304 Epub 2012/09/18. doi: 10.1186/1752-1947-6-304. PubMed PMID: 22978818; PubMed Central PMCID: PMCPMC3469395. 22978818 10.1186/1752-1947-6-304 PMC3469395

[12] Dellinger GW Lynch CA Mihas AA Colonic ganglioneuroma presenting as filiform polyposis. J Clin Gastroenterol 1996;22:66–70. Epub 1996/01/01. PubMed PMID: 8776101. 8776101 10.1097/00004836-199601000-00019

[13] Fernandes A Ferreira AM Serra P Intestinal ganglioneuromatosis: an unusual aetiology for occult gastrointestinal bleeding. BMJ Case Rep 2015;2015 (pii):bcr-2015-211764. 10.1136/bcr-2015-211764 PMC4593261 26424825

